# Effect of angiopoietin-like protein 4 on rat pulmonary microvascular endothelial cells exposed to LPS

**DOI:** 10.3892/ijmm.2013.1420

**Published:** 2013-06-20

**Authors:** YUXI WANG, HAILONG CHEN, HAILONG LI, JINGWEN ZHANG, YANYAN GAO

**Affiliations:** 1 Dalian Medical University, Dalian, Liaoning, P.R. China; 2 Department of General Surgery, The First Affiliated Hospital of Dalian Medical University, Dalian, Liaoning, P.R. China; 3 Department of Central Laboratory, Dalian Municipal Central Hospital, Dalian, Liaoning, P.R. China; 4 Dalton Cardiovascular Research Center, University of Missouri, MO, USA

**Keywords:** angiopoietin-like protein 4, pulmonary microvascular endothelial cell, acute lung injury, F-actin

## Abstract

Pulmonary microvascular endothelial cells (PMVECs) possess highly proliferative and angiogenic capacities and are localized at the critical interface between the blood and microvessel wall; they are the primary targets of inflammatory cytokines during lung inflammation. Angiopoietin-like protein 4 (Angptl4) is a circulating protein that has recently been implicated in the regulation of angiogenesis and metastasis. This study aimed to investigate the effect of Angptl4 on rat PMVECs (RPMVECs) exposed to lipopolysaccharide (LPS). The cell culture was stimulated with LPS. Total Angptl4 cDNA was obtained from Source BioScience. The PCR product was cloned into the pcDNA3.1-eGFP or the pcDNA3.1-eGFP-Angptl4 vector, which were then transfected into the RPMVECs using SuperFect transfection reagent. The Angptl4 mRNA levels, protein levels and cell morphology of the RPMVECs in the experimental groups were detected using real time-PCR, western blot analysis, MTT assay, ELISA and confocal microscopy methods, respectively. The Angptl4 expression vector, pcDNA3.1-eGFP-Angptl4, was successfully constructed. The Angptl4 mRNA level in the LPS-pcDNA3.1-eGFP-transfected group (blank control) was slightly increased and was significantly higher in the experimental group compared with the empty vector and blank control group with significant differences. Pro-apoptotic caspase-8, -9 and Bax protein were inhibited, while p-AKT/AKT and p-MEK1/2 protein expression was also decreased. The rosiglitazone group had significantly decreased levels of the inflammatory cytokine, tumor necrosis factor (TNF)-α (P<0.01). The overexpression of Angptl4 inhibited the LPS-induced increase in the permeability of the RPMVECs, which was associated with the depolymerization of central F-actin in the RPMVECs. In conclusion, our study demonstrates that the overexpression of Angptl4 exerts protective, anti-inflammatory and anti-angiogenic effects. It represents a novel therapeutic target gene for the treatment of acute lung injury induced by LPS.

## Introduction

Angiopoietin-like 4 (Angptl4), one of the key components of the renin-angiotensin system, regulates glucose homeostasis, insulin sensitivity and lipid metabolism (1–4). This multi-functional hormone also affects the proliferation and apoptosis of vascular endothelial cells. A recent study demonstrated that the overexpression of Angptl4 impairs tumor growth associated with enhanced apoptosis, and plays a role in the inflammatory response; however, the mechanisms involved remain unknown (5). Angptl4 expression is upregulated under a variety of conditions, including treatment with glucocorticoids, peroxisome proliferator-activated receptor (PPAR) agonists and transforming growth factor-β (TGF-β) (6–9). Moreover, the expression of Angptl4 is markedly induced under ischemic and hypoxic conditions (10).

Acute lung injury (ALI) is a very common clinical presentation characterized by severe clinical symptoms. Lipopolysaccharide (LPS), a major component of Gram-negative bacterial outer membranes, is an endotoxin which is believed to be the main initiator for the microcirculatory abnormalities in septic ALI (11). It has been reported that Angptl4 is a downstream target gene of the ligand-activated transcription factor, PPAR-γ (12). Studies have shown that PPAR-γ plays an anti-inflammatory role in gastric inflammation induced by ischemia/reperfusion (I/R) (13–17), aspirin (18) and *Helicobacter pylori* infection (19) in rats. The PPAR-γ ligand, troglitazone, has been shown to markedly decrease the severity of pancreatic and pulmonary injury in acute pancreatitis (AP) by reversing the increase in the mRNA expression of the pro-inflammatory cytokines, interleukin (IL)-6 and tumor necrosis factor (TNF)-α, in cerulean-induced pancreatitis in mice (20). The lung microvascular endothelial cell response to sepsis or ALI is incompletely defined, even though they appear to be the first cells of the lungs to be altered during ALI. The responses of lung microvascular endothelial cells lead to changes in the permeability of the vessels. This raises some important issues: i) whether Angptl4 is expressed in rat pulmonary microvascular endothelial cells (RPMVECs); and ii) effects of Angptl4 overexpression that occur during the LPS-induced injury of the pulmonary microvascular endothelium. This may be important in the modulation of endothelial function during LPS-induced ALI. Therefore, in this study, we used RPMVECs to determine whether Angptl4 overexpression attenuates the inflammatory response and whether the regulation of Angptl4 affects the modulation of endothelial function during LPS-induced ALI.

## Materials and methods

### Reagents

LPS from *Escherichia coli* and phalloidin-tetramethylrhodamine B isothiocyanate were purchased from Sigma-Aldrich (St. Louis, MO, USA). Polyclonal antisera against Angptl4, p-MEK1/2, p-AKT, Bax, caspase-8 and -9 were purchased from Santa Cruz Biotechnology, Inc. (Santa Cruz, CA, USA). The Angptl4 gene was purchased from Source BioScience (Nottingham, UK, BC078944). Extracellular matrix (ECM), fetal calf serum, penicillin and streptomycin were purchased from ScienCell Research Laboratories (Carlsbad, CA, USA). Plastic tissue culture flasks were from Costar (Cambridge, MA, USA).

### Isolation of RPMVCs and primary cell culture

The isolation of microvascular endothelial cells was performed according to a modified method originally developed by Chen *et al*(21). Briefly, the fresh lungs isolated from the sacrificed rats were washed with 50 ml serum-free DMEM. The pleura was discarded from the lung tissue and the outer edges of the lung lobe, which did not contain large blood vessels, cut off and minced in serum-free DMEM using scissors. The pellet was rinsed followed by the addition of DMEM containing 20% fetal calf serum, 100 U/ml of penicillin and 0.1 mg/ml streptomycin. The cells were incubated at 37°C in a 5% CO_2_ incubator. After 60 h, the residue lung tissues were removed and the cells were grown in plastic tissue culture flasks. The culture medium was replaced every 3 days and after reaching confluence, the cells were treated with a 0.25% solution of trypsin. The RMVECs were identified according to morphological and functional criteria. They were examined under an inverted microscope by phase-contrast microscopy.

### Experimental design

The cells were randomly divided into the following groups: i) control (CON) group; ii) LPS group: cells were exposed to 100 ng/ml LPS and incubated for 6, 12 and 24 h; iii) LPS + rosiglitazone (ROZ) group: cells were exposed to 100 ng/ml LPS and 50 μg/ml ROZ and incubated for 6, 12 and 24 h; iv) LPS + GW9662 group: cells were exposed to 100 ng/ml LPS and 50 μg/ml GW9662 and incubated for 6, 12 and 24 h; v) pcDNA3.1-eGFP group: cells were transfected with pcDNA3.1-eGFP and incubated for 48 h; vi) pcDNA3.1-Angptl4-eGFP group: cells were transfected with pcDNA3.1-Angptl4-eGFP and incubated for 48 h; vii) LPS + pcDNA3.1-eGFP group: cells were transfected with pcDNA3.1-eGFP and incubated for 48 h; they were then exposed to 100 ng/ml LPS and incubated for 6, 12 and 24 h; viii) LPS + pcDNA3.1-Angptl4-eGFP group: cells were transfected with pcDNA3.1-Angptl4-eGFP and incubated for 48 h; they were then exposed to 100 ng/ml LPS and incubated for 6, 12 and 24 h.

### Angptl4 gene transfection

Cells (3–5 generation) were used for transfection. The day before transfection, cells were seeded at approximately 5×10^5^ cells/well in a 60-mm dish with 2 ml appropriate growth medium until the cells reached 40–80% confluence. The following day, 5 μg pcDNA3.1-eGFP or pcDNA3.1-Angptl4-eGFP DNA were separately dissolved in TE buffer, pH 7–8, with cell growth medium containing no serum, proteins or antibiotics to a total volume of 150 μl. The solution was mixed and centrifuged briefly to remove drops from the top of the tube. Subsequently, 30 *μ*l SuperFect transfection reagent (Qiagen, Hilden, Germany) were added to the DNA solution. The solution was then mixed by pipetting up and down 5 times and the samples were incubated for 5–10 min at room temperature to allow transfection complex formation. Subsequently, 1 ml cell growth medium (containing serum and antibiotics) was added to the reaction tube containing the transfection complexes, after being washed once with PBS. The solution was then mixed by pipetting up and down twice and the total volume was immediately transferred to the cells in the 60-mm dish. The cells were incubated with the transfection complexes for 2–3 h at 37°C in a 5% CO_2_ incubator. The cells were transfected with the pcDNA3.1-eGFP or pcDNA3.1-Angptl4-eGFP constructs and incubated for 24–48 h post-transfection to detect the levels of gene expression.

### Determination of Angptl4 mRNA expression by real-time PCR

Total cellular RNA was extracted from the cells 48 h post-transfection using RNAiso Plus (Takara Bio, Inc., Shiga, Japan) according to the manufacturer’s recommendations. The level of Angptl4 mRNA expression was quantified by real-time PCR using Thermo Scientific Maxima SYBR-Green/ROX q-PCR Master Mix (2X) (Fisher Scientific, Vilnius, Lithuania). RNA was solubilized in RNase-free water and quantified by measurement of the absorbance at 260 nm. The potency was 220–280 ng/*μ*l (the purity of RNA was assured by examining the OD 260/280 as 1.6/1.9). cDNA was synthesized using an iScript™ cDNA synthesis kit and reverse transcription was then performed on a 1 *μ*l RNA sample by the addition of iScript reagents. Amplification and detection were performed using the Rotor Gene 3000™ sequence detection system (Corbett Research, Berkeley, CA, USA) starting with 500 ng of cDNA. The primers and probes used were: Angptl4 forward, 5′-GCCGCTACTATCCACTAC-3′ and reverse, 5′-CCTGTTGCTCTGACTGTT-3′; and β-actin forward, 5′–GGAGATTACTGCCCTGGCTCCTA-3′ and reverse, 5′-GACTCATCGTACTCCTGCTTGCTG-3′. β-actin was used as an internal control. For relative quantification, the copy ratios of Angptl4/β-actin were calculated and used as an indication of the relative expression levels.

### Western blot analysis

After the cell monolayer reached confluence, the RPMVECs were washed with ice-cold PBS and were then resuspended at 1×10^6^ cells/100 μl in ice-cold lysis buffer (50 mmol/l Tris-HCl, pH 8.0, 150 mmol/l NaCl, 1% Triton X-100, 1 mmol/l phenylmethyl sulfonyl fluoride, 0.02% sodium azide and 1 *μ*g/ml aprotinin) and the adherent cells were scraped off the plate into 100 μl lysis buffer/50 cm^2^ culture plate surface. The cell lysates were placed on ice for 20 min and then centrifuged at 12,000 × g for 10 min at 4°C. The post-mitochondrial supernatant fraction was removed and stored in aliquots at −20°C. The protein concentration of the samples was estimated using an ultraviolet spectrophotometer (Perkin-Elmer, Norwalk, CT, USA). Cell lysates were separated by sodium dodecyl sulfate-10% polyacrylamide gel electrophoresis (SDS-PAGE). Briefly, after the SDS-PAGE gels were run, they were transferred onto polyvinylidene difluoride (PVDF) membranes using a semi-dry blot system (Trans-Blot SD Semi-dry Transfer Cell; Bio-Rad, Richmond, CA, USA). The membrane was blocked with blocking buffer (0.4% gelatin) for 1 h at 37°C and then probed with primary antibodies. Dilutions for primary antibodies were as follows: anti-rat p-MEK1/2 antibody (200 *μ*g/ml; 1:400), anti-rat Bax antibody (200 *μ*g/ml; 1:400), anti-rat p-AKT (T450) antibody (200 *μ*g/ml; 1:400, all from Santa Cruz Biotechnology, Inc.), anti-rat AKT antibody (200 *μ*g/ml; 1:400, Bioworld Technology, Minneapolis, MN, USA), anti-rat casapase-8 and -9 antibody (200 *μ*g/ml; 1:400), rabbit polyclonal anti-rat Angptl4 antibody (200 *μ*g/ml; 1:300) and anti-mouse β-actin antibody (200 *μ*g/ml; 1:400, all from Santa Cruz Biotechnology, Inc.), overnight at 4°C. The membrane was washed with PBST (PBS containing 0.5% Tween-20) and then incubated with horseradish peroxidase-conjugated goat anti-rabbit or rabbit anti-goat, rabbit anti-mouse secondary antibodies (500 *μ*g/ml; 1:10,000) at 37°C for 1 h. Following repeated washes with PBST, the antibody-antigen complexes were detected with ECL reagent (Immobilon™ Western Chemiluminescent HRP Substrate; Millipore, Billerica, MA, USA) and exposed to X-OMAT BT film (Kodak). Films were scanned and the optical density (OD) of each band was detected using the ChemiDoc™ XRS+ imaging system (Bio-Rad). The molecular weights of the proteins were estimated by comparison with the positions of the standard.

### Cell viability assay

The viability of the cultured cells was determined by MTT assay. The RPMVECs (10×10^4^ cells/well) were plated in 24-well plates and incubated at 37°C in a 5% CO_2_ incubator. The cells were then divided into the normal, LPS, LPS + ROZ, LPS + GW9662, pcDNA3.1-eGFP, pcDNA3.1-Angptl4-eGFP, LPS + pcDNA3.1-eGFP and LPS + pcDNA3.1-Angptl4-eGFP group and incubated for different periods of time (6, 12 and 24 h). Subsequently, 100 μl of MTT (5 mg/ml) were added to each well for an additional 4 h at 37°C. The supernatant was removed and DMSO was added to dissolve the formazan crystals. The optical absorbance was measured at 540 nm.

### Enzyme-linked immunosorbent assay (ELISA)

ELISA was performed to quantify the concentration of TNF-α in the culture medium using commercially available kits. The TNF-α kit was purchased from R&D Systems (Minneapolis, MN, USA).

### Immunofluorescence

The RPMVECs were washed once with PBS and fixed in 3.7% formaldehyde solution in PBS for 10 min. They were then washed extensively with PBS. They were dehydrated with aceton and then permeabilized by incubation in PBS containing 0.1% Triton X-100. After being washed with PBS; the cells were stained with 50 *μ*g/ml fluorescent phalloidin conjugate solution in PBS for 40 min at room temperature. They were then washed several times with PBS to remove unbound phalloidin conjugate and incubated for 10 min with DAPI. They were then washed extensively with PBS. The stained cells were then examined under a Leica confocal laser scanning microscope (Leica Microsystems, Mannheim, Germany).

### Statistical analysis

All data are expressed as the means ± SD and were analyzed statistically using one-way ANOVA followed by the Newman-Keuls test. A P-value <0.05 was considered to indicate a statistically significant difference. All statistical analyses were performed using Graph Pad Prism 5 software.

## Results

### Characteristics of RPMVECs

**Figure 1:**
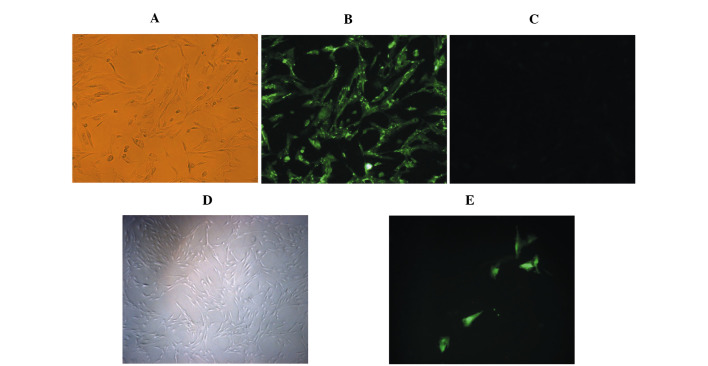
(A and D) Cultured normal rat pulmonary microvascular endothelial cells (RPMVECs). (B) Positive group: RPMVECs were incubated with rabbit anti-rat CD31 antibody and FITC-labeled goat anti-rabbit IgG. (C) Negative group with 5% BSA instead of primary antibody incubation. (E) RPMVECs transfected with pcDNA3.1-eGFP and incubated for 48 h as the blank control group.

The cells grew initially as capillary-like structures and assumed the typical cobblestone morphology of endothelial cells at confluence (Fig. 1A). These cells were characterized as endothelial cells by CD31 antigen expression. Using immunofluorescence, the positive expression of CD31 antigen in the RPMVECs was demonstrated by green particles in the cytoplasm (Fig. 1B). The cells displayed negative staining by BSA (Fig. 1C). The cells were transfected with the pcDNA3.1-eGFP or the pcDNA3.1-eGFP-Angptl4 vector and incubated for 48 h. (Fig. 1E)

### Effect of LPS on Angptl4 mRNA and protein expression in RPMVECs

**Figure 2:**
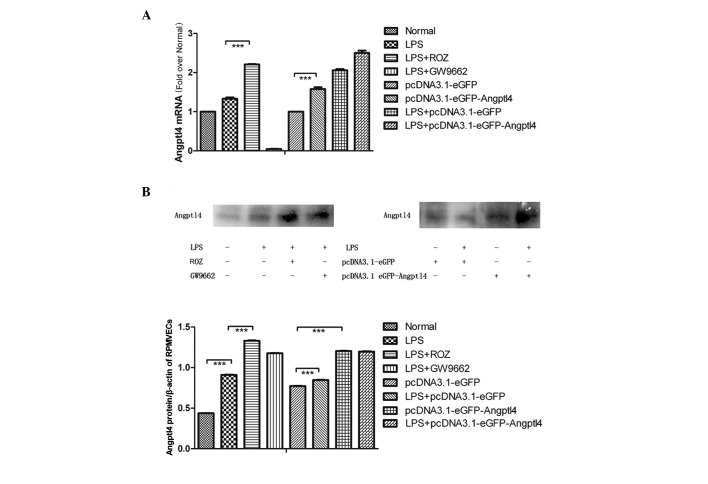
The expression of Angptl4 mRNA was determined by real-time PCR. (A) The results showed that the expression of Angptl4 in the pcDNA3.1-eGFP-Angptl4-transfected group was markedly increased compared with the control group (^***^P<0.01). (B) The protein expression was also altered; however, the protein expression may be influenced by other factors after DNA transcription; there was no difference in protein expression between the LPS-pcDNA3.1-eGFP-Angptl4 group and the pcDNA3.1-eGFP-Angptl4 group (P>0.05). The effects of rosiglitazone [(ROZ) group] were similar to those induced by pcDNA3.1-eGFP-Angptl4 (P>0.05) and differed from the lipopolysaccharide (LPS) group (^***^P<0.01).

The RPMVECs were cultured and exposed to LPS for 24 h and 2 groups of cells were administered ROZ or GW9662. The protein expression of Angptl4 was determined by western blot analysis using Angptl4 antibodies as described in Materials and methods. The mRNA expression of Angptl4 was determined by real-time PCR (Fig. 2A). The mRNA and protein expression of Angptl4 was slightly increased following exposure to LPS. ROZ significantly increased the expression of Angptl4 and GW9662 had the opposite effect. These results indicate that LPS induces a slight increase in the expression of Angptl4 in the PMVECs and ROZ markedly induces its expression.

### Effect of LPS on upregulation of Angptl4 mRNA and protein expression in RPMVECs

The RPMVECs were cultured and transfected with the pcDNA3.1-eGFP or the pcDNA3.1-eGFP-Angptl4 vector and then stimulated with LPS (Fig. 2B). We found that the mRNA levels increased by almost 2-fold compared with the blank group, whereas the levels tripled following treatment with ROZ and LPS. The protein levels were also altered.

### Anti-apoptotic effect of the overexpression of Angptl4 on RPMVECs exposed to LPS

**Figure 3:**
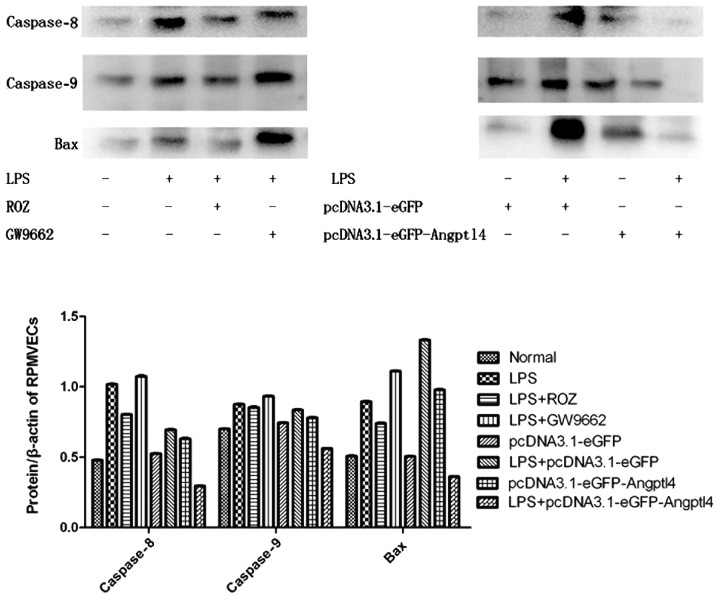
The mRNA and protein expression of the pro-apoptotic factors, Bax, caspase-8 and -9, was markedly decreased in the rosiglitazone (ROZ) and LPS-pcDNA3.1-eGFP-Angptl4 group compared with the lipopolysaccharide (LPS) group (P<0.01). The expression was higher in the transfected groups compared with the control group; this may be due to the harmful effects of transfection.

RPMVECs administered with ROZ and the transfected cells were cultured and exposed to LPS for 24 h. We then detected the expression levels of the pro-apoptotic factors, Bax and casapase-8 and -9 by western blot analysis (Fig. 3). Our results revealed that the expression of these factors was markedly decreased with the upregulation of Angptl4. Of note, an opposite effect occurred after transfection. This may be due to the secretion of Angptl4 protein as a protective factor during acute injury.

### Measurement of TNF-α concentration in culture medium by ELISA

**Figure 4:**
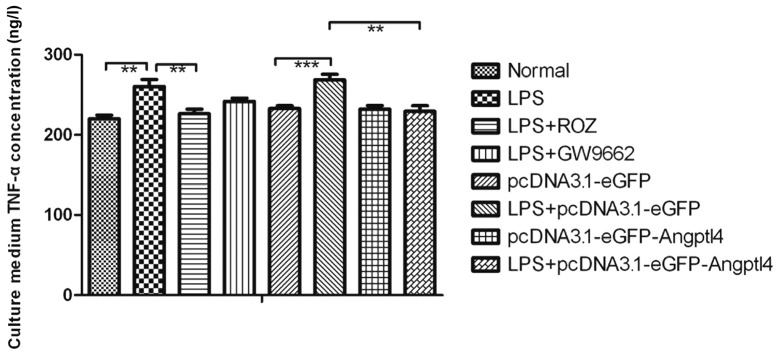
TNF-α concentration in the cell culture medium was examined by ELISA. Data are presented as the means ± SD. ^**^P<0.05, normal vs. lipopolysaccharide (LPS) group. ^***^P<0.01, ROZ vs. LPS + pcDNA3.1-eGFP group. ^***^P<0.01, LPS + pcDNA3.1-eGFP group vs. the pcDNA3.1-eGFP-Angptl4 group.

To determine whether the overexpression of Angptl4 affects angiogenic and inflammatory molecules, such as TNF-α, ELISA was carried out. The TNF-α concentration in the cell culture medium in the LPS group was markedly increased compared with the CON group (Fig. 4, P<0.01). However, the decrease in the TNF-α concentration in the ROZ group and the group transfected with Angptl4 and treated with LPS was significant (P<0.01). These results suggest that the improved anti-inflammatory functions may partly contribute to the beneficial metabolic effects of Angptl4. These data demonstrate that the secretion of Angptl4 in serum may influence the TNF-α concentration; however, the exact mechanisms involved require further investigation.

### Angptl4 overexpression protects RPMVECs against vascular permeability induced by LPS

**Figure 5:**
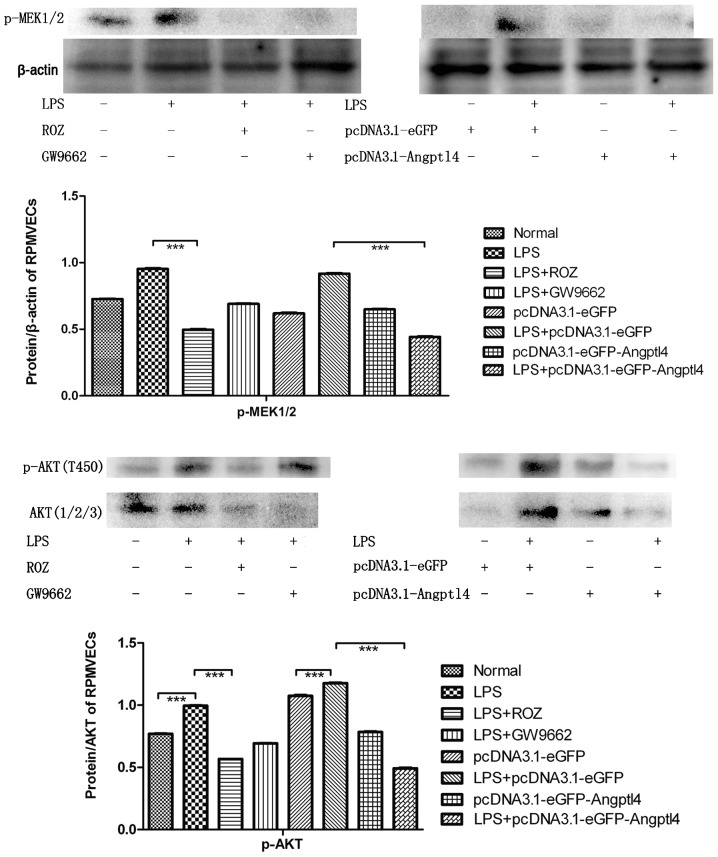
Protein expression of p-AKT/AKT and p-MEK1/2 was decreased following the administration of rosiglitazone (ROZ) and pcDNA3.1-Angptl4-eGFP transfection (^***^P<0.01). We deduced that the overexpression of angiopoietin-like 4 (Angptl4) protects pulmonary microvascular endothelial cells (RPMVECs) by inhibiting the MEK-Raf-MAPK cascade, thus inhibiting inflammation.

**Figure 6:**
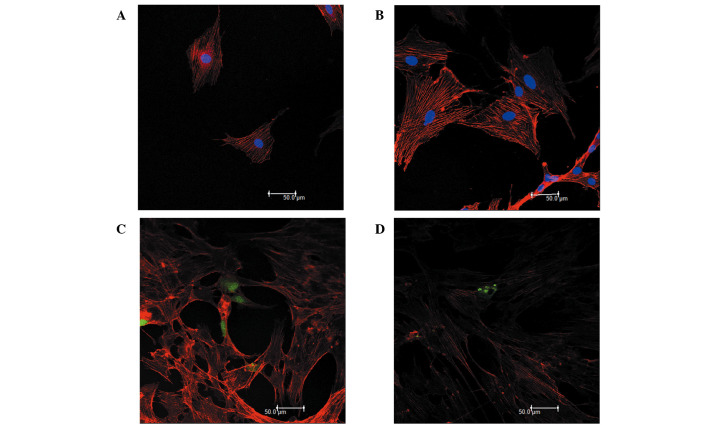
Rat pulmonary microvascular endothelial cells (RPMVECs) were incubated with phalloidin-tetramethylrhodamine B isothiocyanate and then examined under a confocal microscope. (A) Normal group. (B) LPS group. (C) LPS + pcDNA3.1-eGFP group. (D) LPS + pcDNA3.1-Angptl4-eGFP group.

Previously, it was shown that the stimulation of RPMVECs with LPS results in cytoskeletal rearrangement, which can destroy the F-actin cytoskeletan (22,23). Another study reported that the inhibition of the phosphorylation of ERK1/2 attenuates the polymerization of F-actin induced by LPS (24). In the current study, the overexpression of Angptl4 was used to examine the hypothesis that ERK1/2 activation is involved in LPS-induced actin cytoskeletal rearrangement. As shown in Fig. 5, we found that the p-MEK1/2 protein expression was markedly suppressed in the LPS-pcDNA3.1-Angptl4-eGFP group (P<0.01). As shown in Fig. 6, the RPMVECs exhibited a well organized actin cytoskeleton with F-actin fibers crossing the body of the cells and forming a dense filamentous network. The cells exposed to LPS showed a significant reduction in the number of stress fibers and a different pattern of these stress fibers, i.e., they were distributed in the periphery of the cells (Fig. 6B). The RPMVECs pre-treated with ROZ exhibited a morphology and actin stress fibers similar to those of the control cells. Importantly, as shown in Fig. 6D, transfection with Angptl4 blocked the effects of LPS on the actin cytoskeleton. These results indicate that the overexpression of Angptl4 inhibits ERK1/2 activation and plays a key role in actin cytoskeletal changes in RPMVECs induced by LPS.

### Effect of upregulation of Angptl4 on cell viability

**Figure 7:**
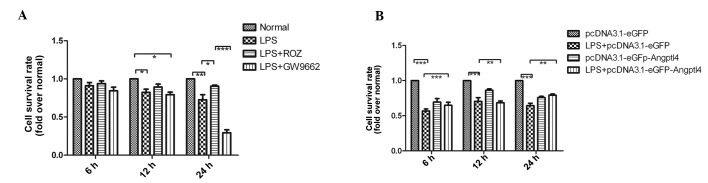
(A) The viability of the cultured rat pulmonary microvascular endothelial cells (RPMVECs) was determined by MTT assay. The results revealed that the administration of rosiglitazone (ROZ) cells prolonged survival in the cells exposed to lipopolysaccharide (LPS) (P<0.01). (B) The viability of cultured RPMVECs was determined by MTT assay. The results revealed that transfection with LPS-eGFP-Angptl4 prolonged survival compared with the blank control (P<0.01).

The effect of the upregulation of Angptl4 on cell viability was dose- and time-dependent (Fig. 7). The absorption values significantly increased following treatment with ROZ (50 *μ*g/ml) and transfection with Angptl4 for 12 and 24 h (P<0.05). The results revealed that the upregulation of Angptl4 improved the cell viability of the RPMVECs exposed to LPS following treatment with ROZ and transfection with Angptl4 for 24 h. Therefore, we selected the time point of 24 h to observe the effects of ROZ on cell apoptosis.

## Discussion

The results of the present study demonstrate the effect of Angptl4 expression on RPMVECs during an acute inflammatory stroke. This process needs to be explored further. The pulmonary endothelium serves as a semi-selective barrier between the plasma and interstitium of circulatory cells, macromolecules and bioactive agents. The maintenance of this semi-selective barrier represents an important physiological process for vessel wall homeostasis and lung function. Injury to the endothelium results in barrier dysfunction with exudation of proteins and fluid within the interstitial tissue and alveolar space that contributes to edema in lung injury.

LPS, a major component of Gram-negative bacterial outer membranes, is an endotoxin that is believed to be the main initiator of microcirculatory abnormalities in septic ALI. The administration of LPS in various models has been shown to induce profound vascular leakage *in vivo*(25) and to increase the permeability of cultured endothelial cells. Studies have shown that in cultured endothelial cells, an LPS-induced increase in endothelial permeability occurs through several pathways, such as endothelial contraction caused by the RhoA-dependent increase in myosin light chain kinase (MLC) phosphorylation (26), reorganization of actin filaments, protein tyrosine phosphorylation (27), etc.. We found that the F-actin cytoskeleton in the RPMVECs in the LPS group was destroyed and that the cell viability was markedly decreased.

In the present study, we aimed to determine the role of Angptl4 in inflammatory responses. Angptl4 is a 50 kDa protein that belongs to the angiopoietin-like family, all of which have a secondary structural organization similar to angiopoietins, including a NH_2_-terminal coiled domain and a COOH-terminal fibrinogen-like domain (28,29). However, Angptl4 does not bind to either the Tie1 or Tie2 angiopoietin receptors (30). Thus, it is currently considered an orphan ligand whose functions differ from those of Angptl1 and Angptl2. Over the last few years, a number of experimental studies have further demonstrated that Angptl4 is involved in key events of tumor growth (5,31). It has been suggested that Angptl4 inhibits VEGF-induced vascular leaks and neoangiogenesis in tumors, while others have shown that Angptl4 hijacks integrin-mediated signaling to maintain an elevated, oncogenic O_2_:H_2_O_2_ ratio and therefore, confers anoikis resistance to tumor cells, suggesting that Angptl4 is an important player in redox-mediated cancer progression (5).

A previous study showed that Angptl4 is a positive acute phase protein whose expression is increased in the liver, heart, muscle and adipose tissue during the acute phase response (32). In this study, we demonstrate that Angptl4 expression increases in RPMVECs administered with LPS during ALI. The mechanism accounting for the increase in Angptl4 expression during the acute phase response is unknown. Angptl4 expression is stimulated by PPARs (33), although previous studies have shown it is unlikely that PPAR activation accounts for the observed effects (34). The present study confirmed the increased expression of Angptl4 at the mRNA and protein level during the early stages of the LPS stimulation of RPMVECs, which increased following the administration of ROZ and transfection with pcDNA3.1-eGFP-Angptl4.

The molecular mechanisms that underlie the observed functional response heterogeneity to Angptl4 in pulmonary macrovascular cells are unknown but presumably involve the differential activation of signals downstream of Angptl4. Since Angptl4 is highly similar to angiopoietins structurally, it is not surprising that human Angptl4, similar to Ang1, has anti-apoptotic effects on endothelial cells, which can lead to the stabilization of newly formed blood vessels. Consistent with this, human Angptl4 has been shown to promote the survival of endothelial cells and blood vessel formation in *in vivo* experimental systems. Of note, Yang *et al*(35) showed that C-Angptl4 attenuates the bFGF-induced phosphorylation of ERK1/2 MAP kinase, but not that of Akt and p38 MAP kinase. In this study, immunofluorescence staining revealed that transfection with Angptl4 and treatment with ROZ and LPS inhibited p-MEK1/2 expression. This suggests the Raf/MEK/ERK cascade inhibited phosphorylated ERK polymerization with F-actin, inhibiting the depolymerization and decreased density of central F-actin in the PMVECs. It stabilized the formulation and protected the cytoskeleton, while the permeability of RPMVECs was decreased significantly. A previous study suggested that Angptl4 prevents metastasis by inhibiting vascular leaks (36). We confer that the overexpression Angptl4 protects the cytoskeleton of RPMVECs and decreases pro-inflammatory cytokine leaks.

Previous studies have suggested the potential pro-angiogenic activity of Angptl4, and data from several independent laboratories have also demonstrated that Angptl4 is a potent anti-angiogenic factor (34,37). Kim *et al*(30) showed that Angptl4 protects endothelial cells from apoptosis through an endocrine action, whereas Oike *et al*(29) showed that Angptl4 inhibits VEGF-induced vascular leaks and neoangiogenesis. In several studies, VEGF has been shown to induce vascular permeability and lead to neutrophil infiltration, even tissue edema. However, Angptl4 has been shown to inhibit the VEGF-induced phosphorylation of ERK1/2, attributed to its specific suppression of the ERK1/2 MAP kinase pathway (35,38).

In addition, we found that p-Akt (T450) decreased following the induction of the overexpression of Angptl4 in the cells stimulating with LPS. Nevertheless, there is a contradicting result *in vitro*; the transfection of Angptl4 may induce apoptopsis, but may inhibit apoptopsis following exposure to LPS, which decreased Bax and caspase-8, and -9 protein expression in this study. The reasons for these conflicting results and the underlying mechanism of Angptl4 activity in the inflammatory response, may be that p-AKT/AKT downregulation may have inactivated the phosphorylation NF-κB and inhibited the downstream production of inflammatory cytokines (39), such as TNF-α. TNF-α is a pro-inflammatory mediator that promotes the adhesion of leukocytes to the endothelium and plays a vital role in the pathogenesis of severe acute pancreatitis (SAP) (40). The increase in tissue and serum TNF-α concentrations correlates directly with the severity of pancreatic damage and inflammation in ALI (41). It can in turn damage the vascular barrier, promoting leaks. As is already known, TNF-α is mediated by a number of signaling pathways, such as the ERK1/2-MAPK and TPK-Ras-MAPK pathways. The decrease in the TNF-α concentration maybe be associated with the inhibition of the Raf/MEK/ERK cascade. In this study, we found that the TNF-α concentration was markedly decreased in the ROZ and pcDNA3.1-eGFP-Angptl4 groups, which suggests that the overexpression of Angptl4 is associated with the anti-inflammatory response.

In conclusion, the data from the present study demonstrate that Angptl4 is a protective secreted protein that acts downstream of other signaling events. Our results provide novel evidence demonstrating that the possible anti-inflammatory mechanisms of Angptl4 involve the protection of the cytoskeleton by inhibiting cell contraction and decreasing the expression of inflammatory cytokines. Further studies are required to fully elucidate the signaling events underlying the anti-angiogenic properties and anti-apoptotic activity of Angptl4.
